# A preliminary study on the diagnostic value of PSADR, DPC and TSRP in the distinction of prostatitis and prostate cancer

**DOI:** 10.1186/s12885-022-09445-z

**Published:** 2022-03-31

**Authors:** Minxin He, Li Wang, Hong Wang, Fang Liu, Mingrui Li, Tie Chong, Li Xue

**Affiliations:** Department of Urology, The Second Affiliated Hospital of Xi’an Jiaotong University, No.157 Xiwu Road, Xincheng District, Xi’an, 710004 Shaanxi China

**Keywords:** Prostate specific antigen decline rate per week, Degree of prostatic collapse, Tissue signal rate of prostate, Prostate cancer, Prostatitis disease

## Abstract

**Background:**

The purpose of this study was to investigate the ability of differential diagnosis of prostate specific antigen decline rate (PSADR) per week, degree of prostatic collapse (DPC) and tissue signal rate of prostate (TSRP) between prostatitis and prostate cancer.

**Methods:**

The clinical data of 92 patients [prostate specific antigen (PSA) > 10 ng/mL] who underwent prostate biopsy in the Department of Urology, the Second Affiliated Hospital of Xi ’an Jiaotong University from May 2017 to April 2020 were reviewed retrospectively. They were divided into two groups, prostatitis group (*n* = 42) and prostate cancer (PCa) group (*n* = 50), according to pathological results. Parameters, like patient characteristics, PSADR, DPC, TSRP and infectious indicators, were compared and analyzed by t test or non-parametric test to identify if there were significant differences. The thresholds of parameters were determined by the receiver operating characteristic curve (ROC), and the data were analyzed to investigate the diagnostic value in distinguishing of prostatitis and prostate cancer.

**Results:**

There were statistical differences in age, PSADR, DPC, TSRP, neutrophil percentage in serum, white blood cell (WBC) in urine and prostate volume between prostatitis group and PCa group (*P* < 0.001, < 0.001, = 0.001, 0.001, 0.024, 0.014, < 0.001 respectively). There was no statistical difference in serum WBC count, serum neutrophil count, monocyte percentage and urine bacterial count between two groups (*P* = 0.089, 0.087, 0.248, 0.119, respectively). Determined by ROC curve, when the thresholds of PSADR per week as 3.175 ng/mL/week, DPC as 1.113, TSRP as 2.708 were cutoffs of distinguishing prostatitis and prostate cancer. When combining these three indexes to diagnose, the accuracy rate of diagnosis of prostatitis was 78.85%, the accuracy rate of diagnosis of prostate cancer was 97.50%. Univariate analysis suggested that PSADR, DPC and TSRP played an important role in differentiating prostate cancer from prostatitis (*P* < 0.05), multivariate analysis suggested PSADR > 3.175 might be good indicators when distinguishing prostate disease with prostatitis (OR = 14.305, 95%CI = 3.779 ~ 54.147), while DPC > 1.113 and TSRP > 2.708 might be associated with a higher risk of prostate cancer (OR = 0.151, 95%CI = 0.039 ~ 0.588; OR = 0.012, 95%CI = 0.005 ~ 0.524, respectively).

**Conclusion:**

The combination of PSADR per week, DPC, and TSRP might be helpful to distinguish prostate cancer and prostatitis, and can reduce unnecessary invasive and histological procedure.

## Background

Prostatic cancer (PCa) is the second most common cancer among males and the fifth most common cause of cancer-related deaths worldwide [1]. Prostate-specific antigen (PSA) is a serine protease produced by normal, as well as malignant, epitheliums of the prostate gland [2]. The blood level of PSA is often elevated in men with prostate cancer, and the PSA test was originally approved by the US Food and Drug Administration (FDA) in 1986 to monitor the progression of prostate cancer in men who had already been diagnosed with the disease. In 1994, FDA approved the use of the PSA test in conjunction with a digital rectal exam (DRE) to test asymptomatic men for prostate cancer. In addition to prostate cancer, a number of benign (not cancerous) conditions can cause a man’s PSA level to rise. The most frequent benign prostate conditions that cause an elevation in PSA level are prostatitis and benign prostatic hyperplasia (BPH) [3]. According to a study, the percentage of PSA reduction can be used to evaluate the probability of receiving other treatments after local treatment, and can help urologists establish appropriate follow-up strategies and patient counseling after local treatment [4]. However, there were patients with PSA < 10 ng/mL were diagnosed as prostate cancer. A study showed that some clinical factors, like PSA, free/total PSA ratio, PSA density (PSA/total prostate volume), positive family history of PCa, and PI-RADS 3 lesion diameter, could predict malignancy in these patients [5]. Moreover, it was reported serum level of gene expression, PIWIL2, could be a beneficial prognostic indicator for Pca particularly for progressed disease [6].

Therefore, imaging examination results are often combined in clinical practice in order to make a more accurate diagnosis. Magnetic resonance imaging (MRI) is the most commonly used imaging modality for early diagnosis and local staging of cancer. MRI may also provide clues to predict the biologic behavior of tumors. Multiparametric MRI (mpMRI) of the prostate includes T2-weighted imaging, DWI, and dynamic contrast-enhanced (DCE) imaging sequences and, therefore, may provide both anatomic and functional information, making it a popular modality in patients undergoing prostate imaging [7]. However, in clinical practice, some benign prostatic diseases such as prostatitis, fibrosis, glandular dysplasia, non-specific granulomatous prostatitis can also show similar characteristics to prostate cancer. Prostatitis, in particularly, is a common disease in urology and tends to occur in the peripheral prostate band. Low signal similar to cancer foci often appears on T2WI [8], which leads to missed diagnosis of early prostate cancer or misdiagnosis of prostatitis as prostate cancer.

Needle biopsy of the prostate is the most reliable diagnostic method for prostate cancer [9]. It is used to distinguish prostate cancer from other diseases that may raise PSA, but its invasive nature has limited its use. Therefore, it is of great clinical value to find effective, non-invasive clinical indicators to differentiate prostate cancer from prostatitis. No studies have combined PSA decline rate (PSADR) per week, degree of prostatic collapse (DPC) and tissue signal rate of prostate (TSRP)rates to differentiate prostate cancer and prostatitis.

In this retrospective study, TSRP, DPC and PSADR of patients undergoing prostate puncture with PSA > 10 ng/mL were statistically analyzed and compared with pathological results, so as to find and evaluate the application value of the above indicators in differential diagnosis before prostate puncture.

## Methods

### Subjects

A total of 92 patients with who were hospitalized due to physical examination findings of undetermined prostate nodules (PSA > 10 ng/mL) in the Department of Urology, the Second Affiliated Hospital of Xi ’an Jiaotong University from May 2017 to April 2020 were retrospectively selected as the study subjects. Blood and urine test were performed at the time of the patient’s visit. The samples were clean midstream urine and the patient didn’t take any medicines that could affect the results before the test. All patients underwent two or more PSA tests before prostate cognitive fusion targeted puncture and then received needle biopsies. The infection indexes, PSADR, DPC and TSRP in blood and urine routine inspections were statistically analyzed. All study subjects had signed informed consent. All the procedures above were under the review of medical ethical committee (NO: 2019079), and all patients have signed informed consents. All experiments were performed in accordance with guidelines and regulations of The Second Affiliated Hospital of Xi’an Jiaotong University.

### Inclusion and exclusion criterion

#### Inclusion criteria

(1) PSA > 10 ng/mL, no urethral catheterization, digital rectal examination, prostate puncture and other operations that may cause PSA increase were performed during the PSA test; (2) the T2-weighted MRI showed abnormal signals; (3) two or more PSA tests were performed within one month.

#### Exclusion criteria

Patients with the following diseases or conditions were excluded (1) incomplete clinical information; (2) other kinds of malignant disease;(3) in acute infection phase (Percentage of neutrophils ≥ 75%).

### Observation indicators

#### TSRP

The corresponding lesion layers were selected from T2-weighted images of prostate MRI, and the signal values of lesion and surrounding prostate tissue were measured by INFINITT imaging system, and the ratio of high signal value to low signal value was calculated.

#### DPC

The lesion level of prostate was selected, and the transverse, longitudinal diametral and actual area of the prostate at this level were measured by the INFINITT imaging system. The presumed prostate area was calculated by the transverse and longitudinal diametral of the prostate (assumed prostate area = π × transverse diameter × longitudinal diameter /4), and compared with the actual area.

#### PSADR

(PSA value at first time—PSA value at second time) × 7/interval days, and the unit was ng/mL/ week.

**Fig. 1:**
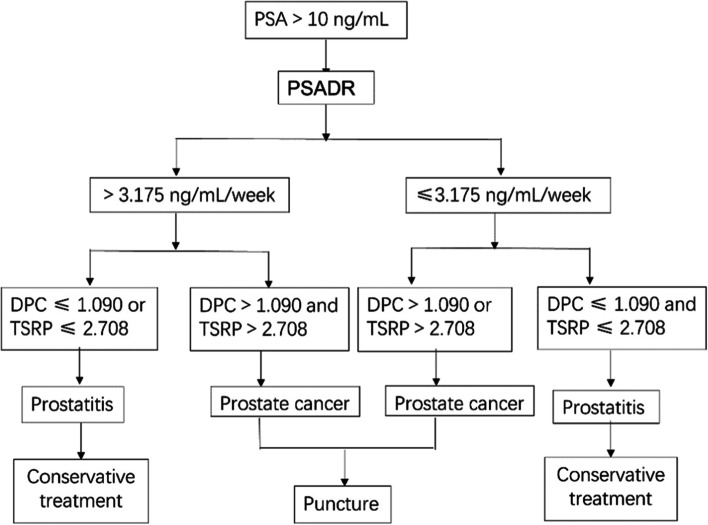
Screening procedures for patients before puncture

The infection indexes, PSADR, DPC and TSRP in PCa group and prostatitis group were compared. The ROC curve was drawn to calculate the area under the curve and to determine the optimal critical value, so as to find its application value in differential diagnosis before prostate puncture. As shown in Fig. 1.

### Statistical analysis

Software SPSS 24 (IBM, New York, USA) was used for Statistical analysis. All the measurement data were expressed as mean ± SD. Kolmogoroc-Smirnov test was performed to analyze the normality. If the data did not meet the normal distribution, median (interquartile spacing) was used for statistical description, and nonparametric test was used for comparison between groups. The measurement data were analyzed by t test or non-parametric test, and the count data were analyzed by χ2 test. The examination and imaging indicators between prostate cancer and prostatic inflammation were compared and analyzed. *P* < 0.05 was considered statistically significant. The receiver operating curve (ROC) was drawn for PSADR, TSRP and DPC, respectively. Then the area under the curve (AUC) was calculated to obtain the optimal critical value. At last, using univariate and multivariate Logistic analysis of PSADR, DPC and TSRP to prove their effect on the differential diagnosis of prostate cancer and prostatitis disease.

## Results

### Patients

There were 50 cases of PCa and 42 cases of prostatitis confirmed by pathology. According to the National Institutes of Health (NIH) classification and definition of the categories of prostatitis are as follows: Category I – Acute bacterial prostatitis (ie, acute infection of the prostate); Category II – Chronic bacterial prostatitis (ie, recurrent urinary tract infection and/or chronic infection of the prostate); Category III: Chronic pelvic pain syndrome (CPPS) A. Inflammatory B. Noninflammatory; Category IV: Asymptomatic inflammatory prostatitis. Since all of these 42 patients lacked typical acute symptoms and the percentage of neutrophils was < 75%, we considered that none of these 42 patients had acute bacterial prostatitis, and they all belonged to chronic bacterial prostatitis, chronic pelvic pain syndrome and asymptomatic prostatitis. In both groups, the mean age of PCa patients was 72.26 ± 1.02 years, and the mean prostate size was 4.9*4.0*4.0 cm (mean volume was 44.15 ± 3.63 cm3). The mean age of patients with prostatitis was 66.52 ± 1.01 years, and the mean prostate size was 5.6*4.9*4.9 cm (mean volume was 77.11 ± 6.60 cm3).

### Clinical characteristics of patients

Clinical characteristics of 92 patients were shown in Table 1. Totally, the average age was 72.26 ± 1.02 years old in PCa and 66.52 ± 1.01 in prostatitis, with *P* < 0.001. In blood routine tests of two groups, only the percentage of neutrophil was significantly different (*P* = 0.024), while there were no significant differences in blood white blood cell count, neutrophil count, blood lymphocyte percentage and blood monocyte percentage (*P* = 0.083, 0.087, 0.050, 0.248 respectively). In routine urine tests, only the difference in urine white blood cell count was significantly different (*P* = 0.014), while the difference in urine bacteria count was not significant (*P* = 0.119).

**Table 1:** Clinical characteristics of the two groups

	PCa	Prostatitis	*t*/Z	*P*
Age (year)	72.26 ± 1.02	66.52 ± 1.01	-3.965	< 0.001
Blood white cell count (× 10^9^ /L)	7.09 ± 0.51	8.23 ± 0.59	-1.701	0.089
Blood neutrophil count (× 10^9^ /L)	4.76 ± 0.47	6.03 ± 0.57	-1.713	0.087
Percentage of neutrophils (%)	64.20 ± 9.72	70.24 ± 2.35	2.217	0.024
Percentage of lymphocytes (%)	26.03 ± 8.14	21.08 ± 2.20	-1.994	0.050
Monocyte percentage (%)	6.63 ± 1.78	6.06 ± 0.44	-1.164	0.248
Urinary white blood cell count (/μL)	128.27 ± 66.34	625.93 ± 387.76	-2.446	0.014
Urinary bacterial count (/μL)	1877.06 ± 1256.44	4152.68 ± 1984.34	-1.560	0.119
Prostate volume (cm^3^)	44.15 ± 3.63	77.11 ± 6.60	-4.276	< 0.001
PSADR (ng/mL/week)	-1.96 ± 1.61	12.14 ± 2.38	-5.926	< 0.001
TSRP	2.61 ± 0.11	2.16 ± 0.10	-3.206	0.001
DPC	1.16 ± 0.02	1.09 ± 0.03	-3.394	0.001
The forecast probability	0.78 ± 0.36	0.27 ± 0.04	-6.490	< 0.001

***Note:**** PSADR* Weekly Decline Rate of PSA, *DPC* Degree of Prostate Collapse, *TSRP* Prostate Tissue Signal Ratio

### PSADR differences between the two groups

**Fig. 2:**
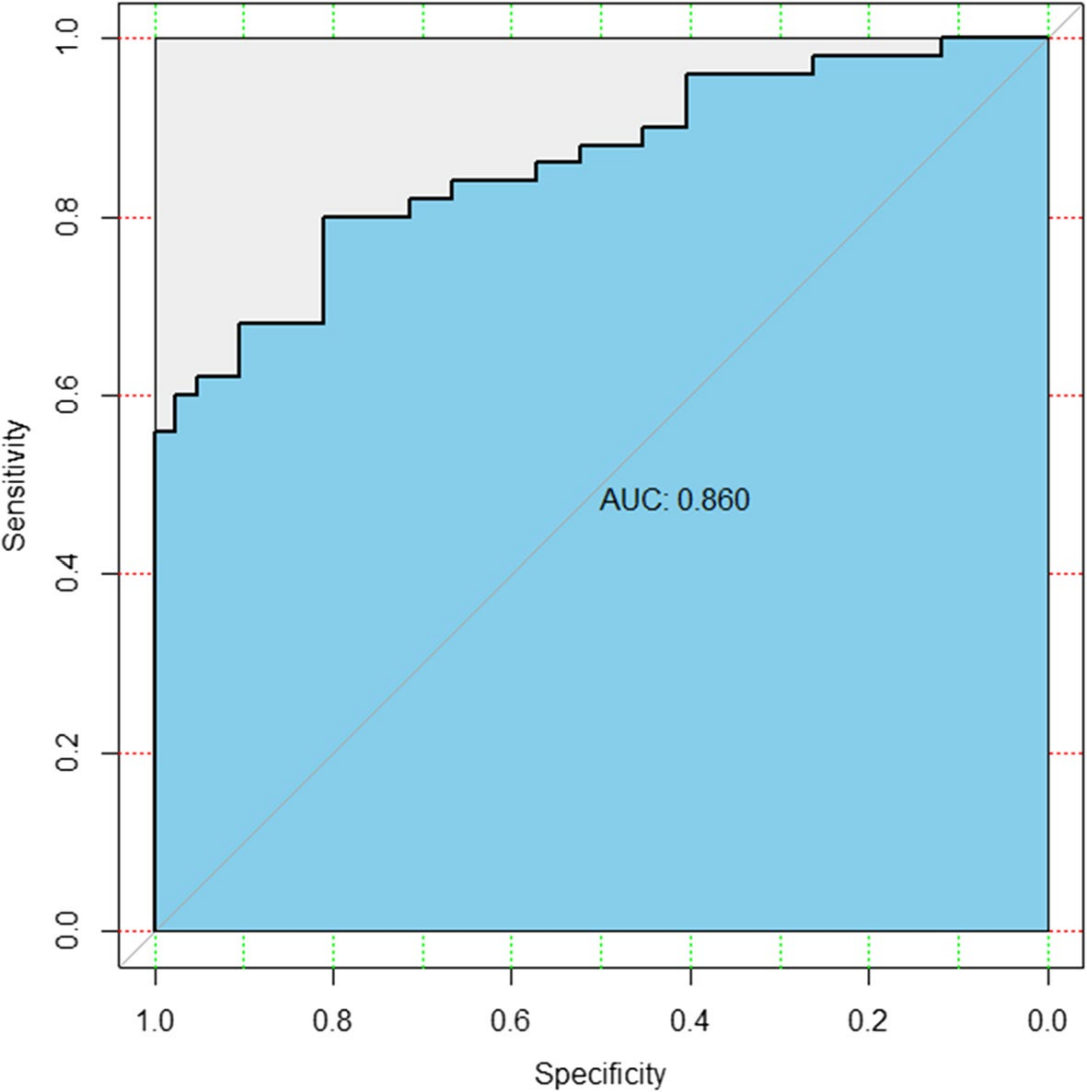
PSADR ROC curve (grey line refers to diagonal reference, and black line is plotted with the true positive rate as the ordinate and the false positive rate as the abscissa.)

Among the data obtained, PSADR in PCa group was lower than that in prostatitis group, and the difference was significant (*P* < 0.001). By drawing ROC curve (Fig. 2, Table 2), it was found that when the critical value was 3.175 ng/mL/week, the Youden index was the largest and the accuracy was the highest. The area under ROC curve (AUC) was 0.860 (sensitivity 80.95%, specificity 80.00%). When PSADR was used to differentiate prostate cancer from prostatitis, the diagnostic coincidence rate of prostate cancer and prostatitis could reach 80.00% and 80.95%, respectively.

**Table 2:** AUC values of PSADR, DPC, TSRP and the combination

	*AUC*	SE	95%*CI*	*P*
PSADR	0.860	0.038	0.786 ~ 0.934	< 0.001
DPC	0.706	0.057	0.595 ~ 0.817	0.001
TSRP	0.695	0.055	0.587 ~ 0.803	0.001
Combination	0.894	0.033	0.830 ~ 0.958	< 0.001

***Note:**** PSADR* Weekly Decline Rate of PSA, *DPC* Degree of Prostate Collapse, *TSRP* Prostate Tissue Signal Ratio, *SE* Standard Error

### DPC differences between the two groups

**Fig. 3:**
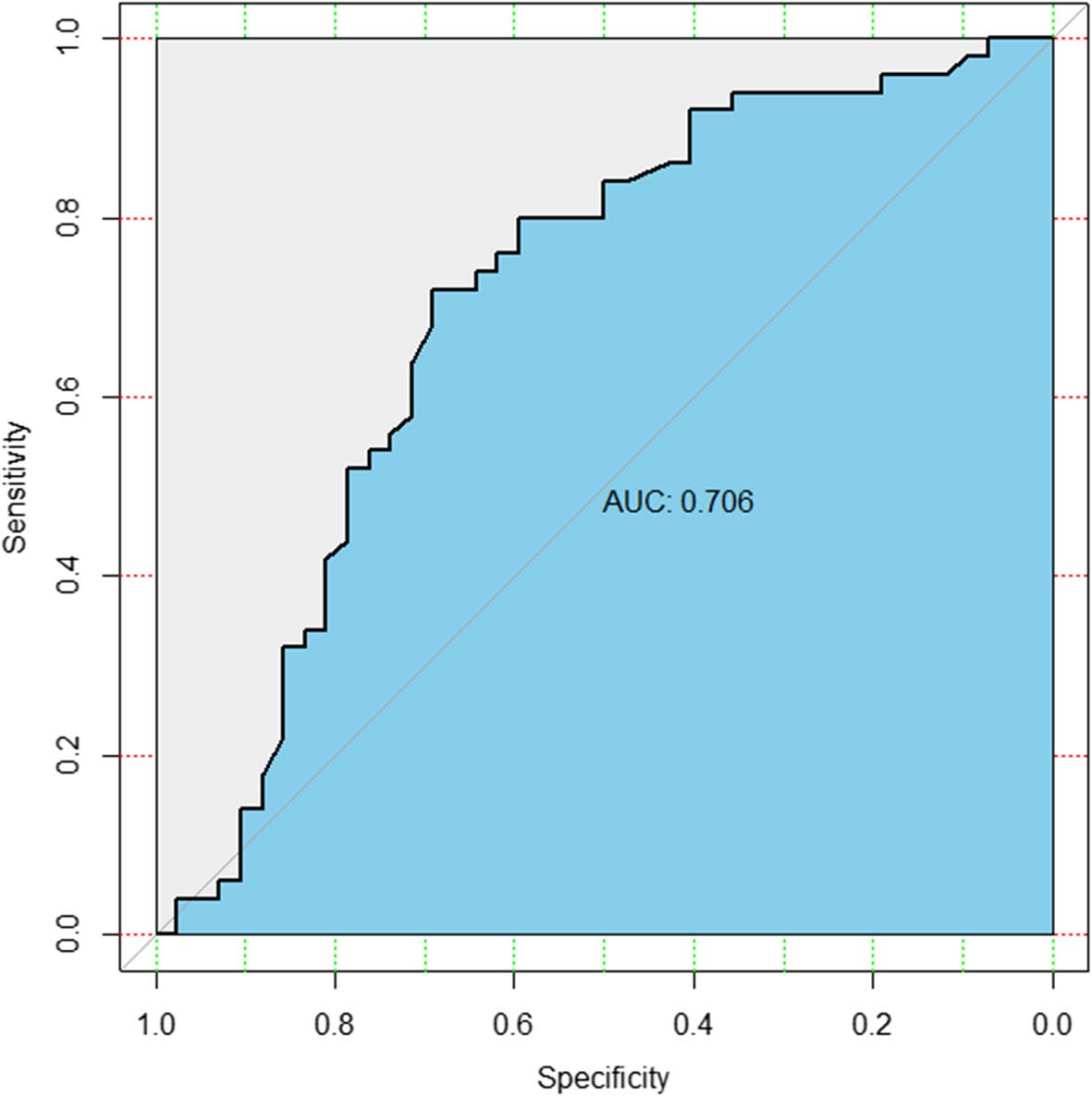
DPC ROC curve (grey line refers to diagonal reference, and black line is plotted with the true positive rate as the ordinate and the false positive rate as the abscissa.)

After analysis, DPC of prostate cancer group was higher than that of prostatitis group, and the difference was significant (*P* = 0.001). By drawing ROC curve (Fig. 3, Table 2), it was found that when the critical value was 1.113, the Youden index is the largest and the accuracy is the highest. AUC was 0.706 (sensitivity 72.00%, specificity 69.05%). When DPC was used to differentiate prostate cancer from prostatitis, the coincidence rate of prostate cancer diagnosis was 72.00%, and that of prostatitis diagnosis was 69.05%, showing high accuracy.

### TSRP differences between the two groups

**Fig. 4:**
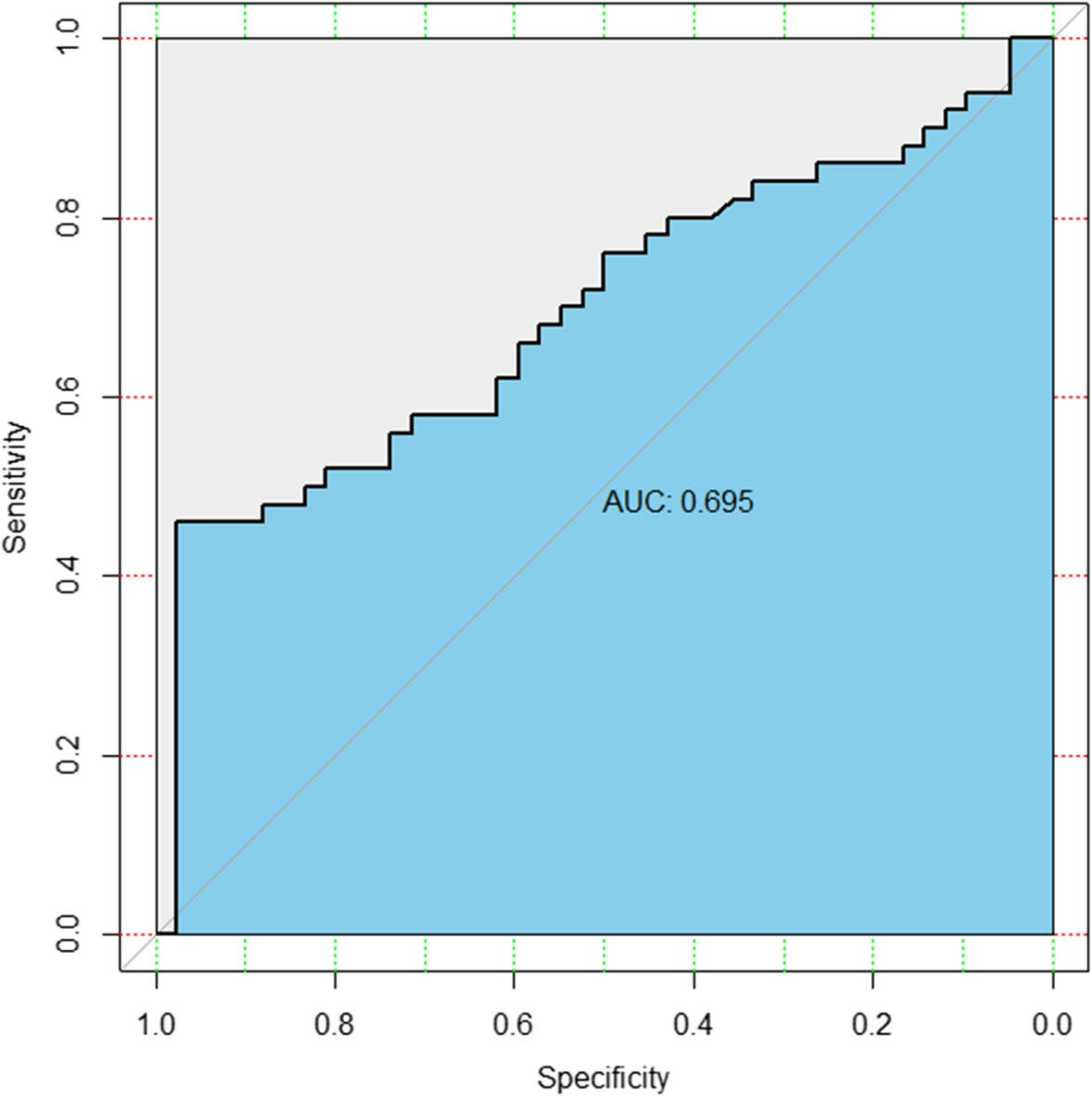
TSRP ROC curve (grey line refers to diagonal reference, and black line is plotted with the true positive rate as the ordinate and the false positive rate as the abscissa.)

Among the 92 valid data, TSRP in prostate cancer group was higher than that in prostatitis group with significant difference (*P* = 0.001). By drawing ROC curve (Fig. 4, Table 2), it was found that when the critical value was 2.708, the Youden index (Youden index = sensitivity + specificity-1) was the largest and the accuracy was the highest. The AUC was 0.695 (sensitivity 46.00%, specificity 97.62%). TSRP was used to distinguish prostate cancer from prostatitis, and the diagnostic coincidence rate of prostate cancer was 46.00% and that of prostatitis was 97.62%, showing high accuracy.

### The combined analysis of PSADR, DPC and TSRP

**Fig. 5:**
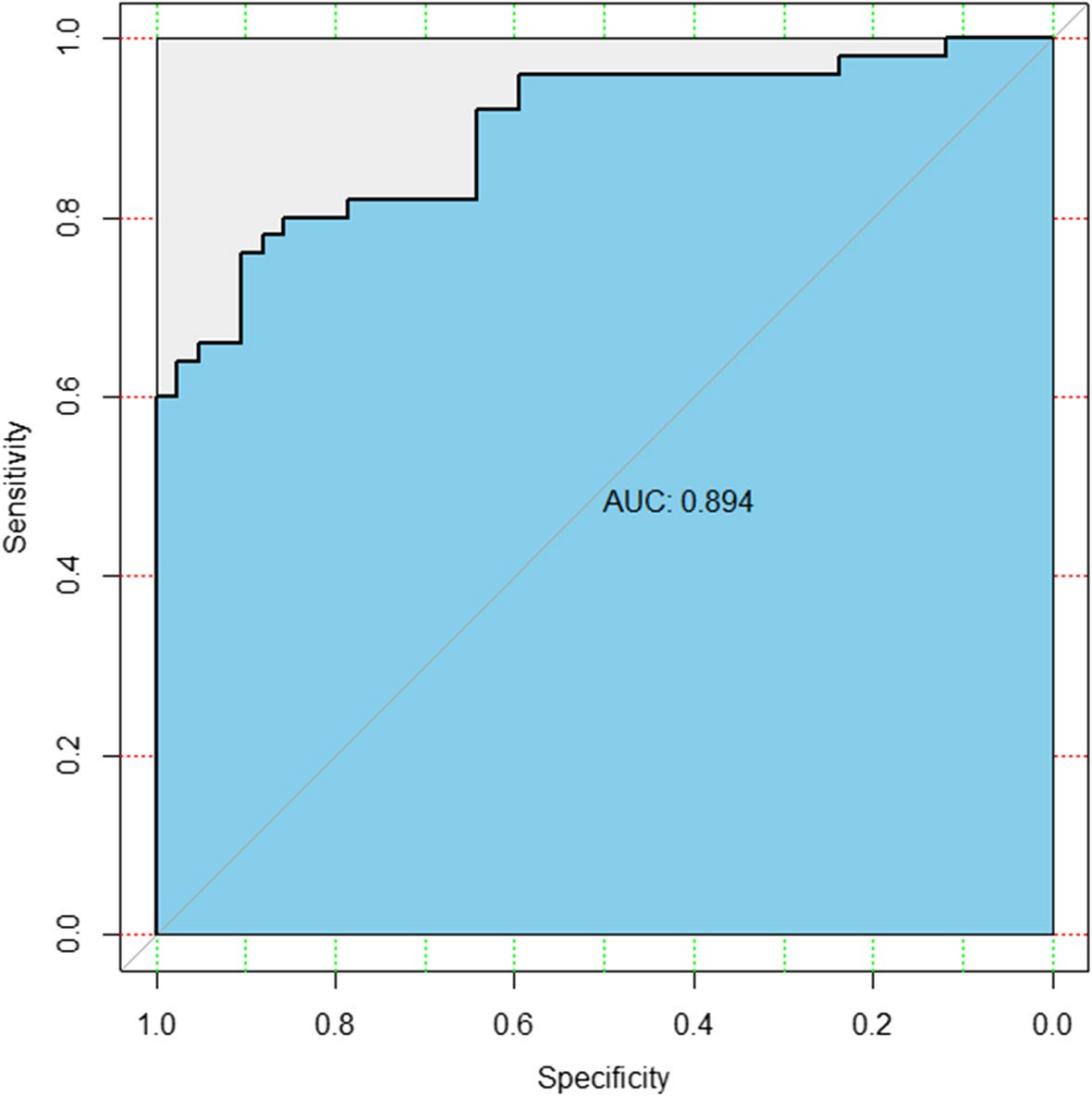
ROC curve of combined PSADR, DPC and TSRP (grey line refers to diagonal reference, and black line is plotted with the true positive rate as the ordinate and the false positive rate as the abscissa.)

When the combination of PSADR, DPC and TSRP was used to distinguish prostatitis disease from prostate cancer, its AUC was 0.894 (Fig. 5, Table 2), showing high accuracy. When the three were combined to distinguish prostate cancer from prostatitis, the coincidence rate of chronic prostate inflammation was 78.85%, and that of prostate cancer was 97.50%.

### Univariate and multivariate analysis of PSADR, DPC and TSRP

Logistic analysis of age, PSADR, DPC and TSRP was conducted (Table 3). We regarded dignoses of patients as the dependent variable, and PSADR ≤ 3.175, DPC ≤ 1.113, TSRP ≤ 2.708 as reference indicator, respectively. PSADR > 3.175, DPC > 1.113, TSRP > 2.708 were used as observation indicator. Univariate analysis suggested that age, PSADR, DPC and TSRP played an important role in differentiating prostate cancer from prostatitis (*P* < 0.001), multivariate analysis suggested PSADR > 3.175 might be good indicators when distinguishing prostate disease with prostatitis (OR = 14.305, 95%CI = 3.779 ~ 54.147), while DPC > 1.113 and TSRP > 2.708 might be associated with a higher risk of prostate cancer (OR = 0.151, 95%CI = 0.039 ~ 0.588; OR = 0.012, 95%CI = 0.005 ~ 0.524, respectively). However, age seemed unable to differentiate prostate cancer and prostatitis.

**Table 3:** Univariate and multivariate Logistic analysis of PSADR, DPC and TSRP

	Univariate logistic analysis			multivariate logistic analysis		
	OR	95%CI	*P*	OR	95%CI	*P*
age	1.130	1.054 ~ 1.212	0.001	1.060	0.975 ~ 1.153	0.169
PSADR	17.000	6.033 ~ 47.906	< 0.001	14.305	3.779 ~ 54.147	< 0.001
DPC	0.174	0.071 ~ 0.429	< 0.001	0.151	0.039 ~ 0.588	0.006
TSRP	0.029	0.004 ~ 0.225	0.001	0.012	0.005 ~ 0.524	0.002

***Note:**** PSADR* Weekly Decline Rate of PSA, *DPC* Degree of Prostate Collapse, *TSRP* Prostate Tissue Signal Ratio

## Discussion

Prostate cancer has a high incidence and death rate, which is a serious threat to men’s health. Prostatitis may mimic PCa because of overlapping clinical, laboratory, and MRI findings. Without histopathologic confirmation, differential diagnosis between these two entities can be difficult [10]. Therefore, early diagnosis and early treatment of prostate cancer patients are of great significance to reduce the burden of national medical insurance and prolong the lives of patients. Currently, screening of PCa mainly relies on PSA and imaging examinations, and prostate puncture and pathology test are still the "gold standard" for PCa diagnosis. However, prostate puncture, as an invasive procedure, has been associated with a number of complications, such as hematuria, rectal bleeding, hemospermia, infection, pain, lower urinary tract syndrome, erectile dysfunction, urinary retention and even death [11]. Therefore, reducing unnecessary prostate puncture is of great significance in clinical practice. In clinical practice, it is found that chronic prostatitis is similar to prostate cancer in laboratory examination, physical examination and imaging examination, and it is the main disease to distinguish it from prostate cancer. However, through literature review, it is found that there is no systematic and comprehensive method and research for the differential diagnosis of these two diseases.

The transient increase of PSA was found in patients with prostatitis, and then the level of PSA could be rapidly reduced to normal after treatment, while the continuous increase of PSA was found in PCa patients with or without anti-infection treatment. Lee, A.G. et al. also found that PSA was unstable in patients with inflammation, which could be significantly reduced after anti-inflammatory treatment [12]. Our study found that prostatitis accounted for 80.95% of 92 patients with PSADR > 3.175 ng/mL/ week. When PSADR < 3.175 ng/mL/ week, the proportion of prostate cancer was 80.00%. It is suggested that PSADR < 3.175 ng/mL/ week might be helpful for the differential diagnosis of prostate cancer and prostatitis, especially for patients with high white blood cell count in urine routine or a history of prostatitis. However, there were studies on the assessment of the PSA in asymptomatic men with elevated PSA and found that PSA changes were not significantly different between patients with prostate cancer and non-cancer patients [13]. Therefore, the effect of single factor assessment on differential diagnosis is not comprehensive, and imaging examination should be performed.

Imaging observations showed that most of the prostate cancer was convex, while most of the prostate inflammatory diseases were capsular atrophy and gland collapse. Prostate cancer is mostly unilateral lesions with focal nodular low signal, clear boundary and mass effect, which often leads to destruction of the prostate capsule [14, 15]. We calculated the oval area as its "ideal prostate area" by measuring the transverse and longitudinal diameters of the prostate, and calculated the DPC compared to its true area. The results showed that when DPC was 1.113, the incidence rate of prostate cancer increased significantly.

The imaging changes of prostatitis and prostate cancer on T2W1 and DWI are similar, expressed as abnormal signal changes in the peripheral zone, but the signal intensity is different [16, 17]. We found that after the signal value of tumor nodules on T2W1 is lower than that in the inflammatory lesions, and the signal value of DWI in tumor nodules can is higher than inflammatory lesions, by measuring the lesions in the signal value of the two groups and the comparison with the surrounding tissue signal values. It was found significant difference in the two groups of signal value. When TSRP < 2.708, prostatitis patients accounted for 97.62%, and when TSRP > 2.708, the proportion of prostate cancer patients was 46.00%.

When PSADR, TSRP and DPC were combined in the differential diagnosis of prostate cancer and prostatitis, the coincidence rate of prostate cancer diagnosis was 97.50%, and the coincidence rate of prostatitis diagnosis was 78.84%. Its AUC was 0.894, showing higher accuracy. Therefore, for patients with PSA greater than 10 ng/mL, it is difficult to distinguish prostate cancer from prostate inflammation by imaging. Differential diagnosis, combined with PSADR, TSRP and DPC, can significantly improve the positive rate of prostate puncture and reduce unnecessary prostate puncture.

Nevertheless, major limitation for this study was that retrospective studies failed to obtain more detailed information and enough sample size. Although MRI was performed on the patients, the MRI findings were not added to this system, which might be investigated in the future study. In addition, as the imaging data viewed by non-professional radiologists, it is inevitable that the data collection is not accurate enough. To verify this conclusion further, we need to expand the sample, conduct prospective study and invite imaging professionals to process collected imaging data.

In conclusion, the combination of PSADR per week, DPC, and TSRP might be helpful to distinguish prostate cancer and prostatitis, and can reduce unnecessary invasive and histological procedure.

## Data Availability

The datasets used and analyzed during the current study are available from the corresponding author on reasonable request.

## Abbreviations

BPH: Benign prostatic hyperplasia
DPC: Degree of prostatic collapse
DRE: Digital rectal exam
PCa: Prostate cancer
PSA: Prostate specific antigen
PSADR: Prostate specific antigen decline rate
ROC: Receiver operating characteristic curve
TSRP: Tissue signal rate of prostate
WBC: White blood cell
